# Osteogenic capillaries orchestrate growth plate-independent ossification of the malleus

**DOI:** 10.1242/dev.123885

**Published:** 2015-11-15

**Authors:** Koichi Matsuo, Yukiko Kuroda, Nobuhito Nango, Kouji Shimoda, Yoshiaki Kubota, Masatsugu Ema, Latifa Bakiri, Erwin F. Wagner, Yoshihiro Takeda, Wataru Yashiro, Atsushi Momose

**Affiliations:** 1 Laboratory of Cell and Tissue Biology, Keio University School of Medicine, 35 Shinanomachi, Shinjuku, Tokyo 160-8582, Japan; 2 Ratoc System Engineering Co., Ltd., 1-24-8 Sekiguchi, Bunkyo, Tokyo 162-0041, Japan; 3 Laboratory Animal Center, Keio University School of Medicine, 35 Shinanomachi, Shinjuku, Tokyo 160-8582, Japan; 4 Department of Vascular Biology, The Sakaguchi Laboratory, Keio University School of Medicine, 35 Shinanomachi, Shinjuku, Tokyo 160-8582, Japan; 5 Research Center for Animal Life Science, Shiga University of Medical Science, Seta, Tsukinowa-cho, Otsu, Shiga 520-2192, Japan; 6 Genes, Development and Disease Group, National Cancer Research Centre (CNIO), Cancer Cell Biology Programme, Melchor Fernandez Almagro 3, Madrid 28029, Spain; 7 X-ray Research Laboratory, Rigaku Corporation, 3-9-12 Matsubara-cho, Akishima, Tokyo 196-8666, Japan; 8 Institute of Multidisciplinary Research for Advanced Materials (IMRAM), Tohoku University, Katahira 2-1-1, Aoba, Sendai Miyagi 980-8577, Japan

**Keywords:** Auditory ossicle, Blood vessel, Synchrotron radiation, AP-1 transcription factor, Osteogenic capillary

## Abstract

Endochondral ossification is a developmental process by which cartilage is replaced by bone. Terminally differentiated hypertrophic chondrocytes are calcified, vascularized, and removed by chondroclasts before bone matrix is laid down by osteoblasts. In mammals, the malleus is one of three auditory ossicles that transmit vibrations of the tympanic membrane to the inner ear. The malleus is formed from a cartilaginous precursor without growth plate involvement, but little is known about how bones of this type undergo endochondral ossification. Here, we demonstrate that in the processus brevis of the malleus, clusters of osteoblasts surrounding the capillary loop produce bone matrix, causing the volume of the capillary lumen to decrease rapidly in post-weaning mice. Synchrotron X-ray tomographic microscopy revealed a concentric, cylindrical arrangement of osteocyte lacunae along capillaries, indicative of pericapillary bone formation. Moreover, we report that overexpression of *Fosl1*, which encodes a component of the AP-1 transcription factor complex, in osteoblasts significantly blocked malleal capillary narrowing. These data suggest that osteoblast/endothelial cell interactions control growth plate-free endochondral ossification through ‘osteogenic capillaries’ in a Fosl1-regulated manner.

## Abstract (precis)

**Summary:** The endochondral ossification of the malleus, an ossicle of the mouse inner ear, occurs around capillaries and is mediated by the AP-1 transcription factor Fosl1.

## INTRODUCTION

Angiogenesis and osteogenesis are closely linked during bone development, remodeling and regeneration (Boerckel et al., 2011; Street et al., 2002; Wan et al., 2010; Xie et al., 2014). From the largest skeletal element (femur) to the smallest (auditory ossicles), most mammalian bones are formed through endochondral ossification. Two crucial components of this process are neovascularization and cartilaginous to bony replacement of extracellular matrix constituents (Ortega et al., 2004). Cartilaginous precursors are initially avascular, but when chondrocytes become hypertrophic, they secrete angiogenic factors such as vascular endothelial growth factor (VEGF) (Gerber et al., 1999; Maes, 2013). This activity in turn stimulates cartilage invasion by capillaries and chondroclasts, which remove cartilage within the perichondrium. Bone formation by osteoblasts then fills the resulting empty space (Maes et al., 2010). Coupling of angiogenesis and osteogenesis during development has been characterized primarily by analyzing the chondro-osseous junction of the metaphyseal growth plate in long bones (Kusumbe et al., 2014). Whereas the highly organized columnar structure of the growth plate facilitates understanding of the morphological features of endochondral ossification (Kronenberg, 2003; Vu et al., 1998), the ossification process of growth plate-free cartilage is not well defined.

The malleus is one of three auditory ossicles in the middle ear, which ossifies without growth plate. Vibration of the tympanic membrane is transmitted to malleus, incus, and stapes, and then to the inner ear. Several characteristics of a short projection of the malleus known as the malleal processus brevis (mPb) make it an ideal system to analyze the non-growth plate type of endochondral ossification. First, the mPb is hemispherical in shape and ossifies without periosteal growth seen in load-bearing long bones (Sharir et al., 2011). Second, its structure is apparently under little functional constraint (Zhang et al., 2003), suggesting that the mPb undergoes a prototypical ossification process. Third, its diameter is ∼300 µm, narrow enough to fit without artificial processing into the field of view of a synchrotron X-ray microscope for high-resolution 3D structural analysis (Nango et al., 2013). Fourth, in mice, the mPb remains fully cartilaginous at birth, and blood vessel invasion and ossification starts only a week after birth (Li et al., 2007), facilitating analysis. Lineage tracing experiments revealed that the mPb originates from the second pharyngeal (branchial) arch (O'Gorman, 2005). This is in contrast with the rest of the malleus, which is derived from the first pharyngeal arch (Anthwal et al., 2013; Chapman, 2011).

Previously, we and others reported that osteoporotic mice lacking osteoprotegerin (OPG) exhibit massive erosion of auditory ossicles by osteoclasts, leading to progressive hearing loss (Kanzaki et al., 2006; Zehnder et al., 2006), and that treatment with anti-resorptive bisphosphonate effectively antagonizes this process (Kanzaki et al., 2009). Conversely, in osteopetrotic mice, in which osteoclastic bone resorption is impaired, the mPb remains fully cartilaginous throughout life (Kanzaki et al., 2011). These studies show that the malleus is subject to bone modeling and remodeling like long bones and vertebrae.

Bone cell differentiation and function are regulated in part by members of the Fos family of transcription factors (Wagner and Eferl, 2005). Genes encoding Fos proteins, which heterodimerize with a Jun family protein to form activator protein (AP)-1, are induced by various stresses in many cell types. Interestingly, mice either lacking or overexpressing specific Fos family factors show diverse bone phenotypes (Grigoriadis et al., 1993; Jochum et al., 2000; Matsuo et al., 2000; Sabatakos et al., 2000). The Fos protein Fosl1 (Fos-like antigen 1, also known as Fra-1, for Fos-related antigen 1; encoded by *Fosl1*) is a known osteogenic transcription factor (Eferl et al., 2004; Jochum et al., 2000) that additionally functions in assembly of endothelial cells into capillary tubes *in vitro* (Evellin et al., 2013). In this study, we assess ossification of the mPb in wild-type mice and in transgenic mice constitutively or inducibly expressing Fosl1. Our findings demonstrate that coordinated bone formation by pericapillary osteoblasts encircling capillaries underlies endochondral ossification in the absence of growth plate, and provide additional insight into spatial coordination of angiogenesis and osteogenesis.

## RESULTS

### Capillary volume in the malleal processus brevis decreases during postnatal development

**Fig. 1.**
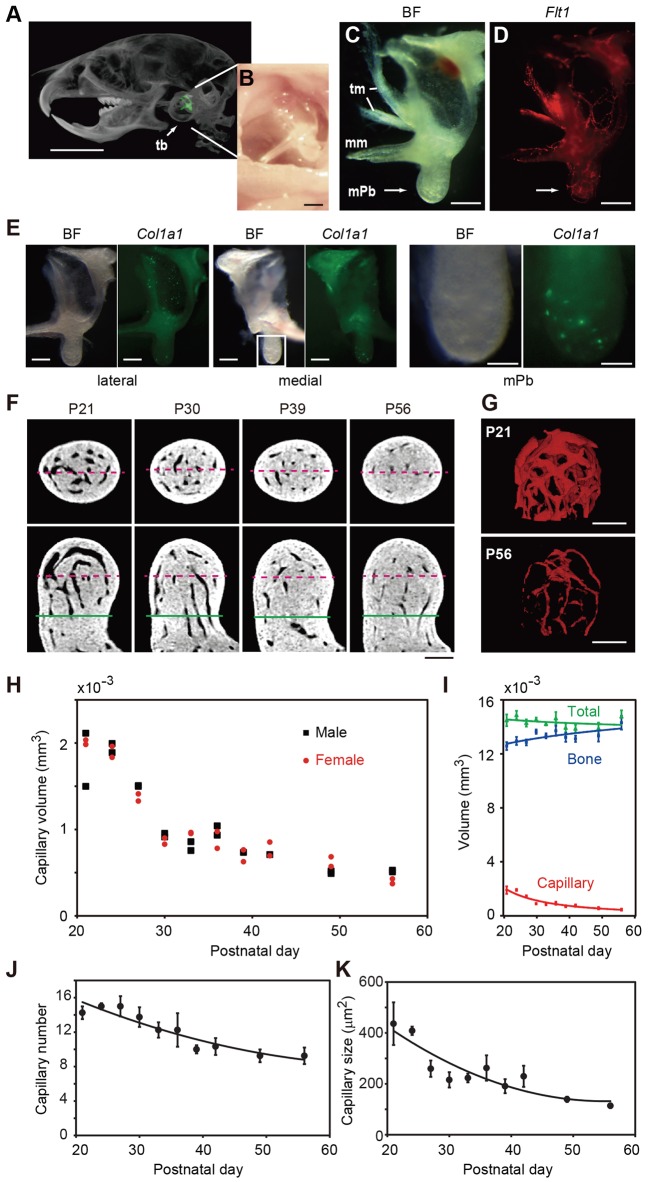
**Reduction in capillary volume in the malleal processus brevis (mPb).** (A) μCT image (voxel size, 40 µm) of the malleus (pseudo-colored green) located in the tympanic bulla (tb) at P21. Scale bar: 5 mm. (B) Malleus appearance after removal of skin and the tympanic membrane. Scale bar: 300 µm. (C,D) Lateral view of the left malleus isolated from a P28 transgenic mouse in bright field (C) and expressing tdsRed driven by the *Flt1* promoter (D). tm, displaced tympanic membrane; mm, malleal manubrium. Scale bars: 300 µm. (E) Lateral and medial view of the left malleus isolated from a P21 *Col1a1*-AcGFP transgenic mouse. Scale bars: 250 µm. Green dots are seen in the mPb (right panel: higher magnification of boxed area in middle panel. Scale bar: 100 µm). (F) Representative μCT images (voxel size, 2.8 µm) of the mPb of wild-type mice. Green lines drawn 300 µm from the distal end of the mPb represent an arbitrary plane of separation between the mPb and of the entire malleus. Red dashed lines indicate positions of reciprocal cutting planes in horizontal (upper) and vertical (lower) views. Scale bar: 100 µm. (G) 3D images of capillaries (pseudo-colored in red) in the mouse mPb at P21 and P56. Scale bars: 100 µm. (H) Distribution of capillary volumes in the mPb of female and male mice from P21 to P56. For each sex, *n*=2 at each time point. (I) Quantification of total volume, and bone and capillary volumes of the mPb in H. (J) Number and (K) cross-sectional area of capillaries were calculated in a plane 150 µm from the distal end of the mPb. Data are represented as means±s.e.m.

The malleus is housed in the tympanic bulla behind the tympanic membrane (Fig. 1A,B). We imaged endothelial cells by red fluorescence in postnatal day (P) 28 mice expressing a tandem red fluorescent reporter (tdsRed) under control of the *Flt1* (FMS-like tyrosine kinase 1, also known as VEGF receptor-1) promoter (Matsumoto et al., 2012). This analysis revealed the presence of blood vessels in the mPb (Fig. 1C,D). To visualize osteoblasts, we generated transgenic reporter mice expressing the *Aequorea coerulescens* green fluorescent protein (AcGFP) under control of the *Col1a1* (collagen, type I, α1) promoter. In the line showing highest AcGFP expression (Fig. S1A-C), the mPb exhibited dots of green fluorescence, suggestive of osteoblast clusters (Fig. 1E).

We next visualized capillaries in the mPb using micro-computed tomography (μCT) at P21, when mice are weaned, and thereafter. At P21, the mPb contained capillaries with large lumens (Fig. 1F). Remarkably, compared with P21 capillaries, P56 capillaries were reduced in diameter, as shown in horizontal and vertical cross sections (Fig. 1F) and in 3D volume renderings (Fig. 1G). In both males and females, capillary volume decreased rapidly during the first two weeks after weaning (P21 to P35) and then more gradually during an additional month (Fig. 1H). During that process, total mPb volume remained largely constant, whereas bone matrix volume increased (Fig. 1I). We conclude that reduced capillary volume was as a result of decreases in both the number and cross-sectional area of capillaries (Fig. 1J,K).

### Osteoblasts and osteocytes are located around narrowing capillaries

**Fig. 2.**
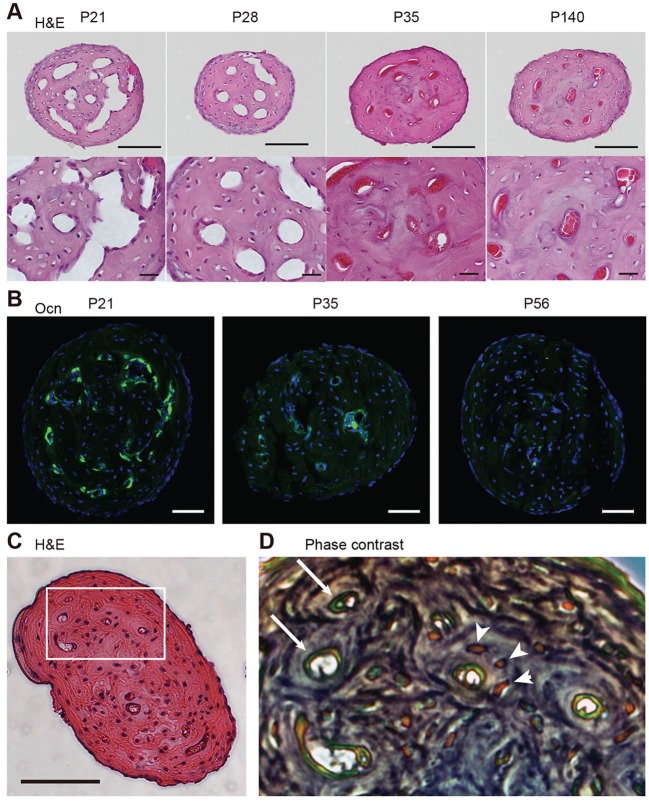
**Histological analysis of the mPb.** (A) Hematoxylin and eosin (H&E) staining of the mPb. Low and high magnifications are shown. Scale bars: 100 µm in upper panels, 20 µm in lower panels. (B) Immunofluorescence staining of the mPb for osteocalcin (Ocn, green). Sections were counterstained with DAPI (blue). Scale bars: 50 µm. (C) Horizontal section showing H&E staining of the mPb from a 12-week-old female mouse. Scale bar: 100 µm. (D) Phase contrast image corresponding to boxed area in C, with green endothelial cells (arrows) and orange osteocytes (arrowheads).

We next examined the mPb in histological sections. Hematoxylin and eosin (H&E) staining of mPb cross sections at P21 revealed a large area containing capillary lumens surrounded by bone matrix (Fig. 2A). At this stage, chondrocytes and cartilage matrix were largely absent from the mPb, as confirmed by an almost total lack of toluidine blue staining of cartilage (Fig. S2). Consistent with μCT observations (Fig. 1F-K), the lumen width within the mPb decreased significantly between P21 and P35. Immunohistochemical staining demonstrated osteocalcin-positive areas in some pericapillary areas, which likely correspond to osteoblast clusters, particularly at P21 and P28 (Fig. 2B). These results show that groups of osteoblasts are located in regions around capillaries. Phase contrast microscopy of a representative H&E-stained section shown in Fig. 2C revealed circular patterns of bone matrix (dark gray) and osteocytes (brown) enclosing capillaries (green) (Fig. 2D), indicative of bone formation around capillaries.

### Osteocyte lacunae are arranged around the capillary loop

**Fig. 3.**
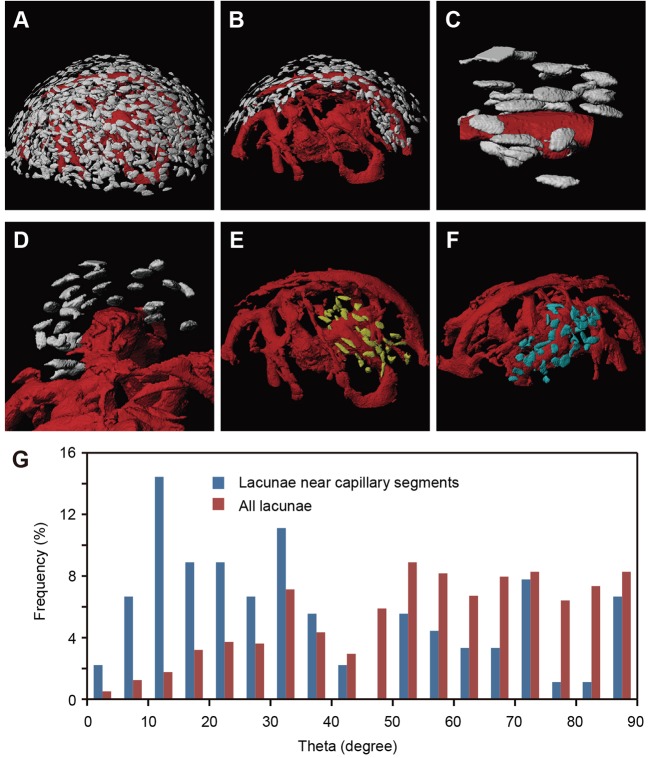
**Osteocyte lacunae align along capillaries in the mPb.** (A) 3D images of osteocyte lacunae and capillaries in the mPb of 16-week-old mice obtained using synchrotron X-ray Talbot tomographic microscopy (voxel size, 0.22 µm). (B) Osteocyte lacunae around a capillary extracted *in silico*. (C,D) Magnified longitudinal (C) and transverse (D) views of a capillary segment and surrounding osteocyte lacunae. (E,F) Osteocyte lacunae (yellow in E and blue in F) around different capillary segments extracted *in silico*. (G) Frequency distribution of angles between longitudinal axes of the capillary shown in B and the orientation of the long axis of osteocyte lacunae near capillaries (blue, *n*=90). Angles between the capillary segment and the long axes of all lacunae shown in A (*n*=967) are shown as controls (red).

To further assess the arrangement of osteocytes around capillaries, we examined osteocyte lacunae in the mPb of adult mice. Synchrotron X-ray tomographic microscopy of the isolated malleus enabled us to visualize both capillaries and osteocyte lacunae in reconstructed 3D images (Fig. 3A). Interestingly, elongated osteocyte lacunae tended to be located along a capillary loop (Fig. 3B,C). Furthermore, we detected concentric distribution of osteocyte lacunae around capillary segments (Fig. 3D). Various capillary segments exhibited similar cylindrical arrangements of osteocyte lacunae along a capillary, indicative of pericapillary bone formation (Fig. 3E,F). To quantify the orientation of osteocyte lacunae in relation to capillaries, we calculated angles between the axes of major capillary segments and each osteocyte lacuna in their vicinity. Most frequently, angles were around 10°, indicating that osteocyte orientation was almost parallel to the orientation of the nearest capillary segment (Fig. 3G). As a control, we calculated angles between the axes of major capillary segments and every osteocyte lacuna in the mPb and observed a more variable angle distribution (Fig. 3G). These data suggest that pericapillary osteoblasts are embedded in bone matrix and elongate along the capillary as they become osteocytes.

### Pericapillary osteoblasts produce bone matrix around capillaries

**Fig. 4.**
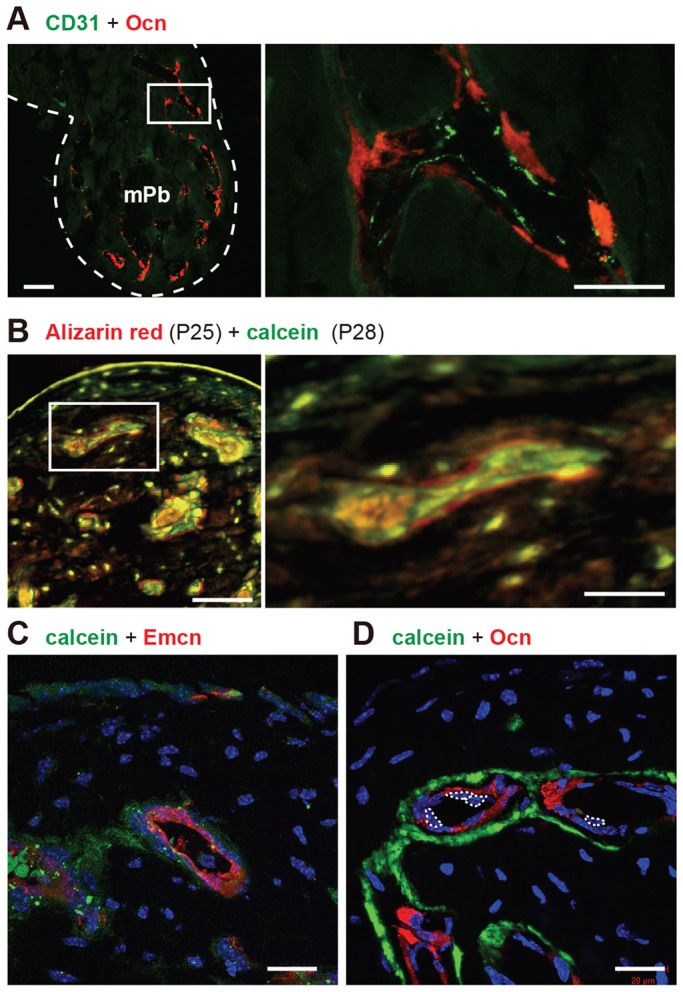
**Bone formation around capillaries.** (A) Immunofluorescence indicating capillary endothelial cells (CD31-positive, green) and osteoblasts [osteocalcin (Ocn)-positive, red] in the mPb at P21. Scale bar: 50 µm. Right panel: higher magnification of boxed area in left panel. White dots outline malleus. Scale bar: 20 µm. (B) Alizarin Red/calcein double labeling of the mPb. Alizarin Red and calcein were injected at P25 and P28, respectively. Mice were sacrificed at P29. Scale bar: 50 µm. Right panel: higher magnification of boxed area in left panel. Scale bar: 20 µm. (C,D) Calcein was injected intraperitoneally at P21 into female C57BL/6 mice, and animals were analyzed 24 h later. Calcein (green) and endomucin (Emcn) (C, red) or osteocalcin (D, red) labeling in the mPb. White dotted lines in D encircle nuclei of endothelial cells. Scale bars: 20 µm.

Immunofluorescence analysis of CD31-positive endothelial cells (green) and osteocalcin-positive osteoblasts (red) further demonstrated that capillaries were surrounded by osteoblasts (Fig. 4A). Moreover, sequential double-labeling of newly formed bone in the mPb using Alizarin Red staining followed by calcein showed that mineralization proceeded inwards around capillaries (Fig. 4B). These data suggest that perivascular osteoblasts lay down osteoid or new bone in an orientation directed away from endothelial cells and that layers of bone matrix surrounding the vasculature rapidly thicken, narrowing the lumen. To visualize mineralizing bone surfaces, we injected calcein intraperitoneally into mice at P21 and performed immunofluorescence analysis 24 h later. Calcein labeling (green) surrounded endomucin-positive endothelial cells (red, Fig. 4C) and osteocalcin-positive osteoblasts, which were located outside of endothelial cells (red, Fig. 4D). These data demonstrate that bone formation occurs around osteogenic capillaries composed of osteoblasts and endothelial cells.

### Fosl1 overexpression antagonizes the decrease in capillary volume in the mPb

**Fig. 5.**
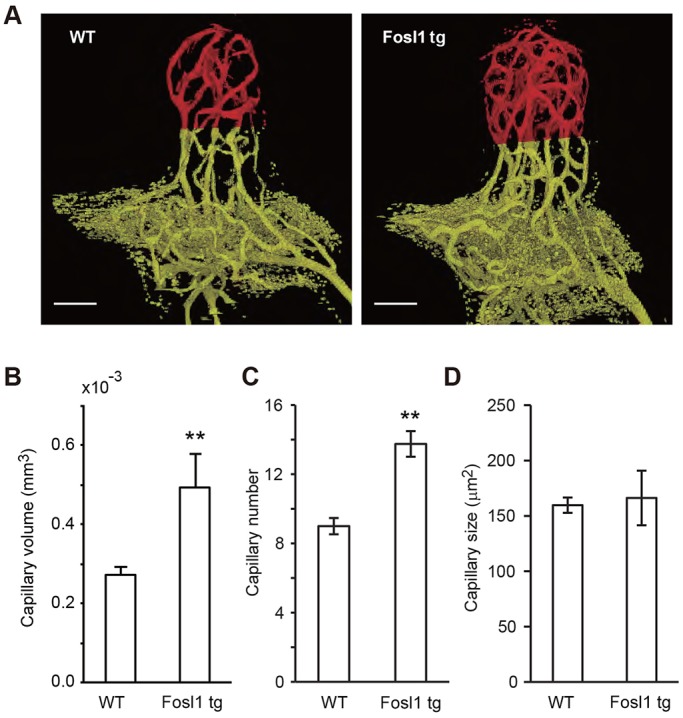
**Constitutive Fosl1 overexpression antagonizes decreases in capillary volume in the mPb.** (A) Constitutive Fosl1 overexpression in H2-K^b^ Fosl1 transgenic mice increases the number of capillaries. Representative μCT images (voxel size, 1.2 µm) of capillaries (pseudo-colored in red). Scale bars: 100 µm. (B-D) Quantitative analysis of changes in capillary volume (B), number of capillaries (C) and cross-sectional area of capillaries calculated in a plane 150 µm from the distal end of the mPb (D). WT, *n*=3; transgenic, *n*=4. **P<0.01. Data are represented as means±s.e.m.

Our observations suggest that capillary endothelial cells might contribute to regulation of pericapillary bone formation. Given that Fosl1functions as an osteogenic factor (Eferl et al., 2004; Jochum et al., 2000), we analyzed capillary volume in transgenic mice constitutively and ubiquitously expressing Fosl1 under control of the H2-K^b^ (H-2 class I histocompatibility antigen, K-B α chain; also known as H2-K1 – histocompatibility 2, K1, K region) promoter (Jochum et al., 2000). Intriguingly, the mPb of these mice showed significantly larger capillary volume relative to wild-type mice (Fig. 5A). When we quantified volume, number and size of capillaries, we found that constitutive Fosl1 expression blocked normal reduction in capillary volume and number (Fig. 5B,C), whereas capillary size was not significantly altered (Fig. 5D).

**Fig. 6.**
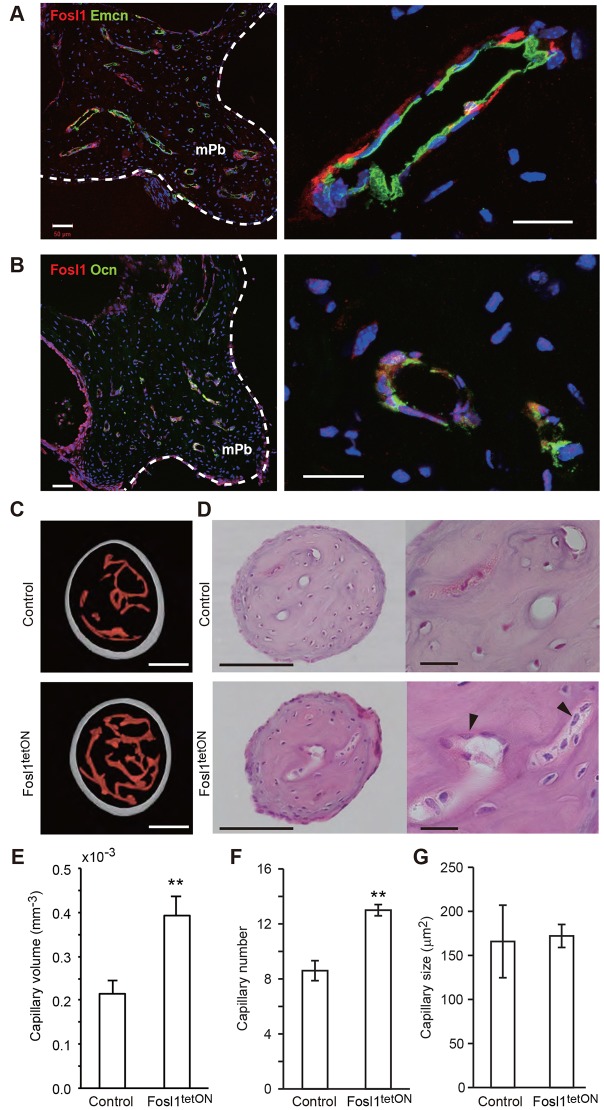
**Conditional Fosl1 overexpression antagonizes normal decreases in capillary volume in the mPb.** Expression of Fosl1 (red) plus endomucin (Emcn) (A, green) or osteocalcin (Ocn) (B, green) in mPb isolated from 4-week-old male Fosl1^tetON^ (*Rosa26*-rtTA;*TetOP*-Fosl1) mice fed food containing dox from P28 to P31. Scale bars: 50 µm in left panel; 20 µm in right panel. White dots outline malleus. (C) Representative μCT images (voxel size, 1.4 µm) of capillaries in the mPb isolated from non-inducible control (*TetOP*-Fosl1, top) and Fosl1-inducible Fosl1^tetON^ (*Rosa26*-rtTA;*TetOP*-Fosl1, bottom) mice. Mice of both genotypes were fed a dox-containing diet starting from P21 and sacrificed at P56. Scale bars: 100 µm. (D) H&E staining of the mPb in non-inducible control and Fosl1^tetON^ mice at P56. Capillary area is greater in Fosl1^tetON^ mice. Arrowheads indicate osteoblasts. Scale bars: 100 µm in left panels; 20 µm in right panels. (E-G) Quantification of capillary volume (E), number (F) and cross-sectional area of capillaries calculated in a plane 150 µm from the distal end (G) of the mPb. Control, *n*=5; Fosl1^tetON^, *n*=4. **P<0.01. Data are represented as means±s.e.m.

We then analyzed inducible Fosl1 transgenic (Fosl1^tetON^) mice (Hasenfuss et al., 2014) after Fosl1 induction for three days from P28 to P31 with food containing doxycycline (dox). Double-immunofluorescence staining revealed that Fosl1-expressing cells surround endomucin-positive endothelial cells (Fig. 6A). The nuclei of osteocalcin-positive osteoblasts were stained with anti-Fosl1 antibody (Fig. 6B), indicating that osteoblasts express Fosl1 in Fosl1^tetON^ mice. Endogenous Fosl1expression in the mPb was also osteoblast-specific, although Fosl1 was expressed at lower levels than in transgenics (Fig. S4), suggesting that transgenic Fosl1 expression mimics endogenous Fosl1 expression in the mPb.

To evaluate phenotypes after a longer period of Fosl1 overexpression, we treated mice with dox-containing food from P21 to P56. This resulted in the appearance of capillaries larger than those seen in non-induced controls, based on 3D renderings (Fig. 6C, Fig. S3). Histological sections revealed abundant pericapillary osteoblasts in mPb of Fosl1^tetON^ mice at P56, suggesting that larger capillary volume did not result from a decreased number of osteoblasts (Fig. 6D). When we quantified capillary volume and number, both were significantly greater in Fosl1^tetON^ mice than in controls (Fig. 6E), whereas capillary size did not differ significantly. These data demonstrate that Fosl1 antagonizes reduced capillary volume and number in the mPb. Collectively, this study reveals that ‘osteogenic capillaries’ exist in the ossifying malleus, a system similar to but clearly distinct from osteons.

## DISCUSSION

In this study, we analyze the ossifying malleus to assess cellular and molecular regulation of angiogenesis and osteogenesis coupling in the absence of a growth plate. We conclude that endothelial cells and osteoblasts interact with each other spatially and functionally in osteogenic capillaries during endochondral ossification.

**Fig. 7.**
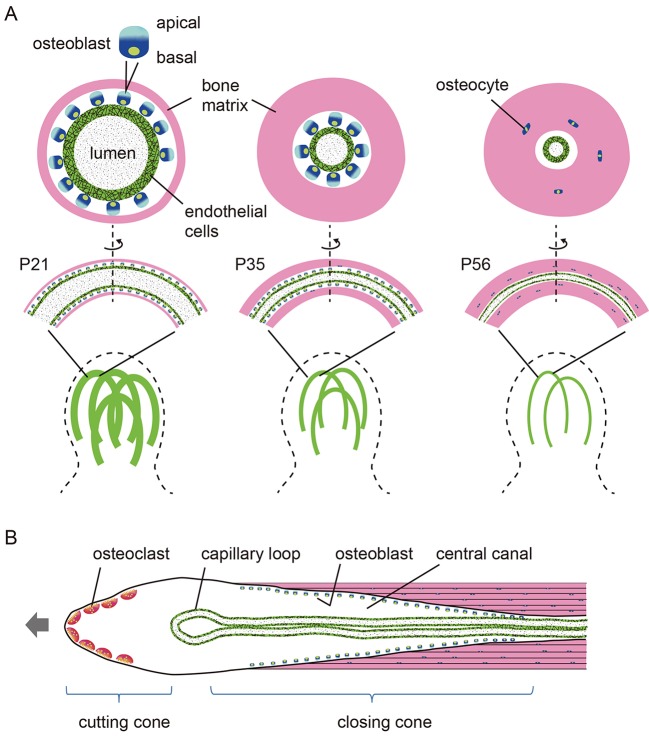
**Comparison of bone modeling and remodeling mediated by ‘osteogenic capillaries’ and osteons, respectively.** (A) Model showing reduced number and size of osteogenic capillaries in the mPb accompanied by bone deposition. Osteoblasts associate with endothelial cells forming a blood capillary (green). Between P21 and P56, the width of vascular lumens (gray stippled) synchronously narrows as a result of formation of perivascular bone (pink) produced by osteoblasts. (B) Scheme showing an osteon, based on Parfitt (1994). Note that the central canal diameter is greatest at the junction of the osteoclast-driven cutting cone and the closing cone, where osteoblasts progressively fill the central canal. Left arrow indicates direction of movement.

At postnatal week three, the mouse mPb exhibits large vascular lumens lined by endothelial cells surrounded by bone matrix. Differentiated osteoblasts are reportedly polarized relative to the site of bone matrix deposition (Izu et al., 2011). In our model, apical surfaces of osteoblasts face the concave surface of preexisting bone matrix, and basal surfaces attach the convex surface of endochondral capillaries (Fig. 7A, P21). We found that osteoblasts located around capillaries within the mPb at this stage are polarized to produce bone matrix away from endothelial cells and that lumen diameter rapidly decreases as bone formation proceeds over the next two weeks (by P35). A fraction of osteoblasts becomes embedded in bone matrix as osteocytes once the vascular lumen narrows (by P56).

High-resolution synchrotron X-ray tomographic microscopy of the mPb also revealed that blood capillaries apparently control the orientation of the longest axis of osteocyte lacuna, which is largely parallel to the orientation of curved capillary segments. This parallel relationship between the central blood vessel and surrounding osteocyte lacunae is reminiscent of osteons, or Haversian systems, in cortical bone of larger mammals, including humans. An osteon consists of a longitudinally oriented central canal surrounded by concentric layers of bone matrix with osteocyte lacunae (Parfitt, 1994) (Fig. 7B). In both osteon-like structures we observe in the mouse mPb and true osteons in cortical bone of larger mammals, osteoblasts fill the tunnel with new bone matrix from the outermost layer inwards towards blood vessels. However, there are differences between these processes. First, osteons are mostly linear and large, reaching 300 µm in diameter (Parfitt, 1994), whereas osteon-like structures in mouse mPb are curved and less than 30 µm in diameter. Second, osteons travel through cortical bone. At their advancing cutting cone, osteoclasts dig a wide tunnel, an event followed by an osteoblastic closing cone that narrows that tunnel, all of which achieves cortical remodeling. By contrast, in curved osteon-like structures in the mPb, pericapillary bone formation occurs synchronously based on developmental time at the same rather than longitudinally changing locations. Third, in osteons, two capillaries and a nerve are located in the central (Haversian) canal (Parfitt, 1994), whereas curved osteon-like structures contain a single capillary. Finally and most importantly, in osteons, endothelial cells are spatially separated from osteoblasts by connective tissue (Parfitt, 1994), whereas we observed direct contact between osteoblasts and endothelial cells in curved osteon-like structures in the mPb (Fig. 7).

A central, single capillary would supply pericapillary osteoblasts with oxygen and raw materials, such as calcium, phosphate and amino acids to make bone matrix, in a process conducive to rapid bone modeling required by a developing animal. It is unclear whether and how direct transportation routes from blood capillaries to bone-forming osteoblasts are established. Recently, association of osteoblastic nestin-positive progenitor cells with the vasculature has been reported in long bones during endochondral ossification (Ono et al., 2014). Moreover, others propose that CD31-high endomucin-high ‘type H endothelial cells’ provide niche signals for perivascular osteoprogenitors in the metaphysis near the growth plate (Kusumbe et al., 2014). Further analysis of the coupling of angiogenesis and osteogenesis is necessary to compare growth plate-dependent and -independent endochondral ossification.

This study focuses on endochondral bone formation after completion of cartilage removal. Cartilage resorption and blood vessel invasion of cartilage anlagen appear to occur concurrently (Karsenty and Wagner, 2002; Maes et al., 2010). In osteopetrotic mice, however, the mPb remains entirely avascular and cartilaginous, indicating that cartilage resorption by chondroclasts is a prerequisite for blood vessel invasion of the mPb (Kanzaki et al., 2011). Factors that coordinate chondroclast polarization and capillary formation in the cartilage matrix required to initiate endochondral ossification remain to be identified in mPb. Identification of molecules cross-talking between endothelial cells and osteoblasts in osteogenic capillaries also awaits further studies. Potential candidates, among many others, include the receptor EphB4 and the ligand ephrinB2, which can regulate blood vessel-wall assembly and osteogenesis (Foo et al., 2006; Wang et al., 2014; Zhao et al., 2006).

We found that broad overexpression of the transcription factor Fosl1, either constitutively or inducibly, antagonized reductions in capillary volume and number in the mPb. The fact that the vasculature phenotype is triggered by post-natal induction of transgene expression suggests that this system remains plastic after embryogenesis. Importantly, Fosl1 induction in Fosl1^tetON^ mice was detected in osteoblasts rather than in endothelial cells, indicating that osteoblastic expression, not Fosl1 expression by endothelial cells or osteocytes, is crucial for the mPb phenotype. Further analysis using mice lacking Fosl1 (Eferl et al., 2004) should shed further light on Fosl1 or AP-1 function in osteoblast–endothelial cell interaction during endochondral ossification. Nonetheless, the Fosl1 function reported here appears noncanonical, as osteogenesis promoted by Fosl1 overexpression should result in the opposite phenotype, namely narrowing of vascular lumens resulting from increased bone formation (Jochum et al., 2000). We did not detect altered auditory brain stem responses (ABR) in transgenic mice constitutively expressing Fosl1, indicating that they can hear (data not shown).

Fosl1 overexpression in osteoblasts reportedly skews expression of bone matrix proteins (Eferl et al., 2004; Nishiwaki et al., 2006). Fosl1 transcriptional targets include modulators of angiogenesis such as plasma membrane plasminogen activator, urokinase (Plau) and its receptor (Plaur) (Kustikova et al., 1998), matrix metalloproteinases (Luo et al., 2010), inflammatory mediators (Yamaguchi et al., 2009) and integrins (Evellin et al., 2013). One possibility is that capillaries associated with osteoblasts expressing Fosl1 might persistently function as ectopic ‘osteogenic capillaries’, thereby accounting for both enhanced bone formation seen in adult transgenic mice (Jochum et al., 2000) and the mPb phenotype reported here.

In summary, we found that the volume of blood vessels in ossifying bone is initially substantial and then rapidly decreases as bone matrix is laid down around vessels, a process regulated by expression of the AP-1 transcription factor Fosl1. Understanding ‘osteogenic capillaries’ and coordinated interaction between osteoblasts and endothelial cells should provide deeper insight into endochondral ossification. Such knowledge could be useful for regenerative medicine or developmental engineering strategies in which cartilaginous anlagen serve as transient structures to regenerate bone tissues (Scotti et al., 2013).

## MATERIALS AND METHODS

### Mouse strains

Generation of H2-K^b^-Fosl1 and *Flt1*-tdsRed transgenic mice was previously described (Jochum et al., 2000; Matsumoto et al., 2012). Doxycycline (dox)-inducible Fosl1-expressing (Fosl1^tetON^) mice were produced by crossing *Rosa26*-rtTA transactivator mice with mice carrying a dox-inducible Fosl1 allele (*TetOP*-Fosl1) (Hasenfuss et al., 2014). Both control and Fosl1^tetON^ mice were fed a diet containing dox at 200 mg/kg (5TP7, PMI Nutrition International). To generate *Col1a1*-AcGFP transgenic mice, a *Sma*I-*Not*I AcGFP cDNA fragment was obtained from the pAcGFP-1 vector (Clontech) and subcloned into the *Not*I site of modified pNASSβ, which contains the 2.3 kb osteoblast-specific mouse *Col1a1* promoter region (Liu et al., 2001). A *Nar*I-*Sal*I fragment containing the *Col1a1* promoter-AcGFP-poly A signal was isolated and microinjected into pronuclei of fertilized eggs from B6C3F1 (C57BL/6×C3H/He) female mice. Three independent lines were obtained. Whole mount fluorescence was visualized using a Leica M205FA stereomicroscope. All mice were bred and maintained under specific pathogen-free conditions. All experiments were approved by the Keio University Institutional Animal Care and Use Committee and were conducted in accordance with Institutional Guidelines on Animal Experimentation at Keio University.

### Histological analysis

Bones were isolated and fixed with 4% paraformaldehyde/PBS overnight. After decalcification in 20% EDTA at 4°C for 48 h or Kalkitox (Wako) for 4 h, hematoxylin and eosin (H&E) staining was performed on 5 µm paraffin sections, which were analyzed microscopically (Axiovert 135, Ziess; BX53, Olympus). For fluorescent double labeling, mice were intravenously injected with 25 mg/kg Alizarin Red (Sigma) at P25 and 2.5 mg/kg calcein (Wako Pure Chemicals) at P28. Mice were sacrificed at P29, and bones were fixed in 4% paraformaldehyde and embedded in methylmethacrylate. Sections of 5 µm were observed by confocal microscopy (FV-10i, Olympus). For immunofluorescence, fresh-frozen mallei were cryosectioned at 6 µm thickness using an adhesive film according to the Kawamoto method (Kawamoto, 2003) on a cryostat (CM3050S, Leica). Anti-mouse CD31 (PECAM-1) hamster monoclonal antibody (1:500, 2H8, Millipore), anti-mouse Fosl1 rabbit polyclonal antibody (1:50, N-17, Santa Cruz), anti-mouse osteocalcin polyclonal antibody (1:1000, ALX-210-333, Enzo), anti-mouse osteocalcin rat monoclonal antibody (1:200, R21C-01A, Takara), and anti-mouse endomucin rat monoclonal antibody (1:50, V.7C7, Santa Cruz) were used. Antigen retrieval was performed for anti-endomucin and anti-osteocalcin antibodies by treatment with 0.01 M citrate buffer (pH 6.0) at 37°C for 45 min, and 20 µg/ml proteinase K treatment at room temperature for 5 min, respectively. Multiple slices per field were evaluated using a maximum intensity projection method on a laser scanning confocal microscope (LSM710, Zeiss).

### Micro-CT analysis

Images (voxel size, 40 µm) were obtained using the R_mCT2 μCT (Rigaku) operated at 90 kV, 160 µA and 512 projections/360°. Images (voxel size, 2.8 µm [1.4 µm]) were obtained using the TDM1000 μCT (Yamato Scientific) operated at 90 [40] kV, 30 [125] µA and 800 [1200] projections/360°. High-resolution CT images (voxel size, 1.2 µm) were obtained using the nano3DX X-ray microscope (Rigaku) operated at 40 kV, 30 mA, 360 projections/180°.

### Synchrotron radiation X-ray CT imaging

A monochromatic X-ray beam (9.0 keV) at the beamline BL20XU of SPring-8 (for Super Photon ring-8 GeV) (Hyogo, Japan) was used with a Talbot phase-sensitive X-ray tomographic microscope operated at 25 s/projection, 1000 projections/360°, as previously described (Nango et al., 2013; Takeda et al., 2008). X-ray imaging optical magnification was 20.2, and voxel size was 0.22 µm

### Image processing

3D data were analyzed using Tri/3D-BON (Ratoc System Engineering), IMARIS 6.3.1 (Bitplane) and ImageJ 1.48v software (National Institutes of Health). Bi-level images were generated based on threshold CT values to extract capillaries and osteocyte lacunae. The direction of the long axis of both segments of blood capillaries and osteocyte lacunae was estimated using the ellipsoid approximation method. The angles between a segment of capillary and the long axes of osteocyte lacunae were calculated from the inner product of two vectors.

### Statistics

Statistical analysis was performed using Student's *t*-test. Data are shown as means±s.e.m. *P* values less than 0.05 were considered statistically significant. *P<0.05, **P<0.001.
